# Hospital-based COVID-19 registry: Design and implementation. Colombian experience

**DOI:** 10.1016/j.mex.2023.102056

**Published:** 2023-02-03

**Authors:** Sarita Rodriguez, Tania M. Guzmán, Eric Tafurt, Estefanía Beltrán, Andrés Castro, Fernando Rosso, Sergio I. Prada, Virginia Zarama

**Affiliations:** a Fundación Valle del Lili, Centro de Investigaciones Clínicas, Cali 760032, Colombia; b Fundación Valle del Lili, Department of Emergency Medicine, Cali 760032, Colombia; c Fundación Valle del Lili, Infectious Diseases Service, Cali 760032, Colombia; d Universidad Icesi - Facultad de Ciencias de la Salud Cali, Colombia; e Universidad Icesi, Centro PROESA, Cali, Colombia

**Keywords:** SARS-CoV-2, COVID-19, Registry, Data, Hospital-based COVID-19 registry

## Abstract

Registries are essential to providing valuable clinical and epidemiological decisions. Designing a registry is challenging because it is time-consuming and resource-intensive, particularly in low- and middle-income countries. Here, we described our experience with the rationale, design, and implementation of a hospital-based COVID-19 registry in Cali, Colombia. We designed and implemented a hospital-based registry over a dynamic web-based structure to record all sociodemographic, clinical, and laboratory tests, imaging, treatment, and outcomes of SARS-CoV-2. We included 4458 confirmed COVID-19 cases of 18 years and older from March 2020 to March 2021. The median age was 48 years. The most frequent comorbidities were hypertension, obesity, and diabetes. The ICU admission rate was 19%, and the in-hospital mortality rate was 20%. The implemented strategies provided rapid and reliable information collection for the registry of emerging studies from the different clinical areas. Regular data quality and feedback are essential to ensure the reliability of the information. The integration of automatic data extraction reduces time consumption in information gathering and resources.

## Graphical abstract

**Figure:**
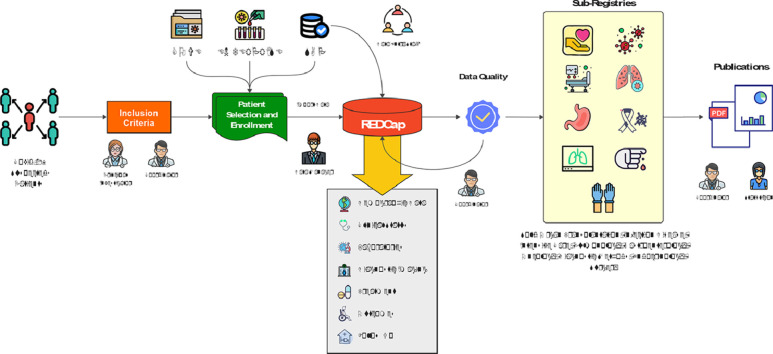

Specifications table

**Table:** 

Subject area:	Medicine and Dentistry
More specific subject area:	Infectious diseases and technological tools
Name of your protocol:	Hospital-based COVID-19 registry
Reagents/tools:	Not applicable
Experimental design:	Not applicable
Trial registration:	Not applicable
Ethics:	Our work did not involve animal experiments, the collection of data from social platforms, or interventions on humans
Value of the Protocol:	• To contribute to the literature on the rationale, design, and implementation of the COVID-19 registry. • To describe the data collection methodology and the data quality control process. • To describe the registry implementation as a helpful resource for understanding the characteristics, clinical outcomes, and impact of COVID-19 in our population.

## Description of protocol

Registries are essential to providing valuable clinical and epidemiological decisions. Designing a registry is challenging because it is time-consuming and resource-intensive, particularly in a low- and middle-income countries. We described the experience and challenges on the rationale, design, methodology, and implementation of RECOVID-19, a COVID-19 registry, in a tertiary healthcare center in Cali, Colombia.

## Study design and population

FVL is the main referral hospital for the country's southwest region, located in Cali, a city with a population of 2.2 million that provides healthcare services for 828,352 people per year (Fundación Valle del Lili, 2020). The initial installed capacity of the hospital was 625 beds, 396 in a general ward and 229 in ICU. Due to the contingency created by SARS-CoV-2, a new isolation ward was established in the Emergency Department, with 10 ICUs and 38 general ward beds (Fundación Valle del Lili, 2020). The first admission of a SARS-CoV-2 infection case was on March 14, 2020, and the first patient record was included on March 27, 2020.

## Registry governance and purposes

A multidisciplinary scientific committee composed of 2 emergency physicians, two infectious diseases specialists, one intensive care specialist, one epidemiologist, and one statistician was responsible for the defining the Registry's purposes, writing the study protocol and the engaging of the stakeholders from FVL and the Centro de Investigaciones Clínicas (CIC). The health information management division provided an internal advisory to assure confidentiality, interoperability, data quality, and security.

The study protocol was approved by the Biomedical Ethics Committee (IRB/EC No. # 1566/8). We followed the ethical principles for medical research outlined by the Declaration of Helsinki and the declaration 8430 of 1993 from Colombia's Ministry of Health. This observational study has no risk for participants, and Informed consent is not required according to the 8430-national resolution.

The implementation of this Hospital-based COVID-19 registry approach was to provide rapid and reliable information on the sociodemographic, clinical, laboratory tests, imaging, treatment, and outcomes of patients with SARS-CoV-2 infection. Furthermore, we proposed:

• Helping clinical and administrative decision-making in real-time through systematic analysis.
• Contributing to the national and international scientific literature on COVID-19 characterization.
• Promoting the creation of sub-registers from different clinical areas to expand the research opportunities.
• Determining incidence, prevalence, and mortality related to SARS-CoV-2 infection in our population.
• Determining sociodemographic and clinical characteristics, laboratories, imaging, treatment, and outcomes.
• Evaluating the association of baseline clinical characteristics with the risk of complications and progress to severe infection in patients during the 28-day hospitalization follow-up.
• Assessing the risk factors associated with a severe COVID-19 clinical presentation.
• Assessing the association between the different treatments and clinical outcomes.
• Comparing clinical outcomes of patients with COVID-19 among the peaks and waves of the pandemic in Colombia.

## Implementation process

### Case definition

All adults (>=18 years) with confirmed SARS-CoV-2 infection by viral real-time RT-PCR test Assay, viral antigen detection, or presence of SARS-CoV2 antibodies with high clinical suspicion treated at FVL. Outpatient was defined as those patients discharged in less than 24 h. Patients with previous diagnosis and resolved SARS-CoV-2 infection were excluded.

### Data collection

The feasibility of data collection was evaluated based on the information availability, accessibility, and gathering time. A comprehensive data set was approved through a broad discussion among the multidisciplinary scientific committee. Afterward, we tested the first version of the data collection forms through a pilot; thus, we evaluated the feasibility, new information requirements, and adjustments. The final version of the COVID-19 Registry was grouped into three data collection forms: admission, follow-ups, and outcomes (Table 1). We created a web-based registry using Research Electronic Data Capture (REDCap) hosted at the FVL data center, which is a secure, web-based software platform designed to support data capture for research studies (Harris et al., 2009, 2019).

**Table 1:** Data set summary.

Type of data	Variables
Sociodemographic & epidemiological	Age, gender, date of admission, home city, healthcare worker, date of symptoms onset, epidemiological link
Medical history	Hypertension, coronary disease, cancer, obesity, DM, pulmonary disease, cerebrovascular disease, chronic liver disease, chronic kidney disease, autoimmune disease, HIV, TB, smoking, organ transplantation, home oxygen requirement, medications
Clinical manifestations	Fever, cough, expectoration, dyspnea, nasal congestion, odynophagia, fatigue, myalgia, arthralgia, anorexia, dysphagia, abdominal pain, diarrhea, nausea, vomiting, chest pain, palpitations, taste alterations, smell alterations, somnolence, headache, hemoptysis, among others.
Physical examination	Vital signs, weight, height, BMI, dyspnea
Diagnostic tests	Confirmed SARS-CoV-2 infection by Reverse transcription-polymerase chain reaction, SARS-CoV-2 antigen test, or antibodies
Laboratory & imaging	Electrocardiogram, chest X-ray, echocardiogram, chest computed tomography, lung ultrasound, blood count, electrolytes, renal function, liver function, ferritin, LDH, CPK, procalcitonin, blood gasses, coagulation times, C-reactive protein, dimer D, fibrinogen, troponin I.
Severity assessment	Asymptomatic, mild disease, moderate disease, severe disease, critical illness, ARDS, sepsis, septic shock, type of admission
Treatment	Steroids, anticoagulants, vasopressors, inotropic
Complications	Renal replacement therapy, myocarditis, cardiogenic shock, VAP
Outcomes	Days of hospitalization, days of IMV, HAI, co-infection, thrombotic events, arrhythmias, discharge status (alive/dead), destination (home, referral, homecare).

^IMV: Invasive mechanical ventilation, HAI: Healthcare-Associated Infections, VAP: Ventilator-associated pneumonia, DM: diabetes mellitus, HIV: human immunodeficiency virus, TB: tuberculosis,^^BMI: Body mass index, LDH, lactate dehydrogenase, CPK: Creatine phosphokinase, ARDS: acute respiratory distress syndrome^.

An active retrospective and prospective search for suspected and confirmed cases was conducted from the daily FVL reports by a physician. The data entry staff carried out daily systematic data collection. Sociodemographic and clinical information was obtained from the patient electronic health record managed by mySiss® SAP system and the diagnostic tests and laboratory from the enterprise platform. The worst available values were recorded in the database for vital signs and blood gasses, among other variables.

All hospitalized patients were followed up prospectively through days 1, 2, 3, 4, 5, 7, 14, and 28 days from admission. All data regarding physical examination, laboratory and images, and disease progression were recorded on each follow-up form.

Different strategies were proposed to avoid errors or omissions in the inclusion of data. One of them was to reduce the number of variables with text fields and include more variables with drop-down and checkboxes format. Furthermore, different tools of the REDCAP platform were used to build data quality rules; thus, the data entry staff were aware of recording errors in real-time. These restrictions included ranges of values, required fields, and the highlight of recorded data inconsistent with the inclusion criteria.

Moreover, special rules were implemented to automatically complete fields, such as age calculation, clinical scores (i.e.,CALL SCORE), and clinical indexes (i.e., PaFi). Finally, all the involved personnel was selected from the full-time FVL staff in the medical field (intern, resident, general practitioner). They received comprehensive prior training on the information requirements and the managing of the RedCap database.

The quality data was performed on a weekly basis. By applying a random sampling of 10% of the total number of records, an exploratory analysis was performed by a statistician. A report of inconsistencies and missing data was generated to be resolved by the data entry staff.

Due to the large number of patients infected by the SARS-COV-2 virus, especially in the different waves of the pandemic, measures were implemented to improve data collection speed, data quality, and cost reduction. For this purpose, automation was implemented in the abstraction of sociodemographic data, laboratory results, medications, vital signs, and length of hospital stay from the patient's electronic medical record in SAP, thus integrating the data into the EDC (RedCap).

### Statistical analysis

A descriptive statistical analysis from the collected date from the first year (Fig. 2) of the pandemic was performed. Categorical variables are presented as frequencies and proportions. Quantitative variables were reported as mean, standard deviation, or median with interquartile range, according to the normality assumption. Analysis was performed using STATA statistical software (Stata Corp, 2011, Stata 12 Base Reference Manual, College Station, TX, USA).

## Protocol validation

**Fig. 1:**
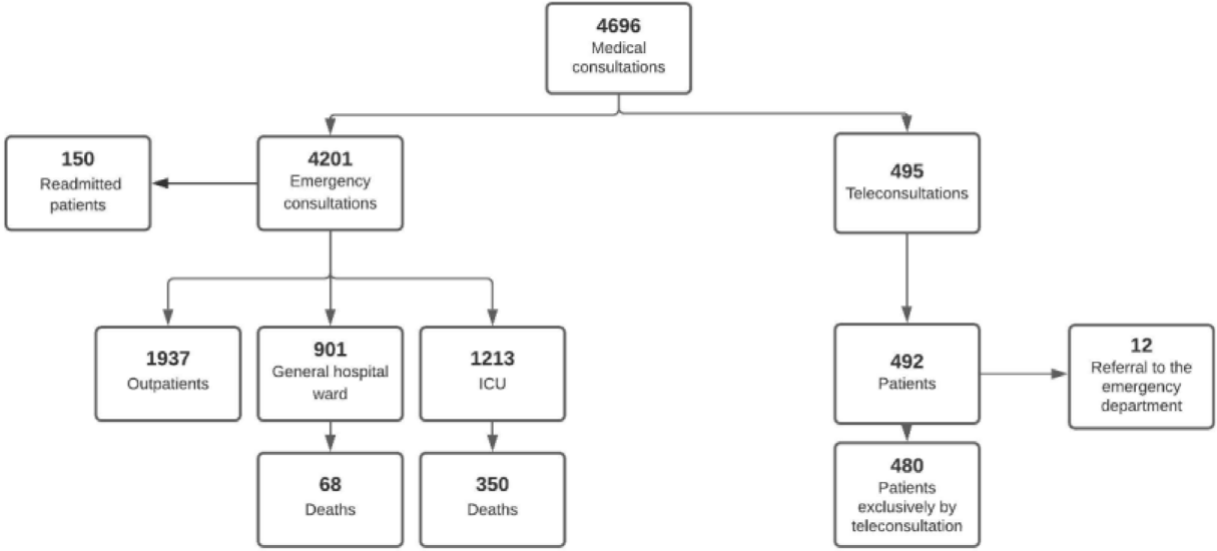
Flowchart of COVID-19 patients included in the study.

The implementation of RECOVID-19 enabled the ease of collection and follow-up of 4696 patients during the first year of the pandemic in a single center. Fig. 1 shows the flow of care for confirmed cases of SARS-CoV-2 infection treated between March 2020 and March 2021.

The median age was 48, with male prevalence (51% vs. 49%). Of all patients, 18.4% were healthcare workers (Median age =31 [IQR = 27 – 38]). Our patients' most frequent baseline medical conditions were overweight, 46.6%, and arterial hypertension, 26%. In-hospital mortality during this period was 20% (418 deaths /2114 hospitalized) and was higher in men (66% vs. 44%).

This Registry has facilitated rapid access to updated and quality information for COVID-19 research. One of the most important outcomes of the Registry is the creation of nine sub-registries that allow the development of multiple research projects in short periods. At the administrative level, we provide updated information regarding the number of suspected and confirmed cases (i.e., outpatient, inpatient, ICU) to facilitate cost and economic analysis, resource planning, and quality and detail on the proper completion of valuable clinical, sociodemographic, and epidemiological data through a collaborative and ongoing communication process.

A systematic approach to data collection of COVID-19 provides valuable information for health policymakers, researchers, and clinical specialists 24. The RECOVID-19 data set included a large number of variables in comparison to other registries worldwide to commit to comprehensive information [1], [2], [3], [4], [5], [6]. In addition, follow-ups give patient monitoring during the hospital stay up to 28 days from admission, which is relevant for a better understanding of the infection course, complications, short-term outcomes, and the potential association of prognostic factors for recovery or worsening of patients with COVID-19.

Even though RECOVID-19 is a single-center registry, we included a more significant number of patients than previous studies reported [4,5,2]. From the design stage, the definition of roles facilitated the completion of specific activities, such as the group of data entry, study coordination (SC), data quality, and analysis. Consequently, during the implementation phase, it was possible to identify the need for additional personnel due to the increase in the number of patients at the national level.

RECOVID-19 is a helpful resource to comprehend the characteristics and impact of COVID-19 during the first year of the pandemic in our population and the basis for ongoing and further research projects. This COVID-19 registry approach provides updated and quality data regarding managing COVID-19 patients and could become a reference for other research groups based on this experience. To our knowledge, this is the first comprehensive Registry report of COVID-19 in Colombia. In summary, our work provides an initial approach to the rationale, design, and implementation of a COVID-19 registry according to structure, data collection methods, and systematic quality process, which is fundamental to generating reliable information.

## CRediT authorship contribution statement

**Sarita Rodriguez:** Software, Validation, Formal analysis, Investigation, Data curation, Writing – original draft, Writing – review & editing, Visualization, Supervision, Project administration. **Tania M. Guzmán:** Investigation, Data curation, Writing – original draft, Writing – review & editing, Visualization, Supervision. **Eric Tafurt:** Investigation, Data curation, Writing – original draft, Writing – review & editing, Visualization, Supervision. **Estefanía Beltrán:** Investigation, Data curation, Writing – original draft, Writing – review & editing, Visualization, Supervision. **Andrés Castro:** Methodology, Formal analysis, Investigation. **Fernando Rosso:** Conceptualization, Investigation, Supervision. **Sergio I. Prada:** Conceptualization, Investigation, Supervision. **Virginia Zarama:** Conceptualization, Investigation, Supervision.

## Declaration of Competing Interest

The authors declare that they have no known competing financial interests or personal relationships that could have appeared to influence the work reported in this paper.

## Data Availability

The data that has been used is confidential.
